# Somite-Derived Retinoic Acid Regulates Zebrafish Hematopoietic Stem Cell Formation

**DOI:** 10.1371/journal.pone.0166040

**Published:** 2016-11-18

**Authors:** Laura M. Pillay, Kacey J. Mackowetzky, Sonya A. Widen, Andrew Jan Waskiewicz

**Affiliations:** 1 Department of Biological Sciences, University of Alberta, Edmonton, Alberta, T6G 2E9, Canada; 2 Neuroscience and Mental Health Institute, University of Alberta, Edmonton, Alberta, T6G 2E9, Canada; 3 Women & Children’s Health Research Institute, University of Alberta, Edmonton, Alberta, T6G 2E9, Canada; Texas A&M University, UNITED STATES

## Abstract

Hematopoietic stem cells (HSCs) are multipotent progenitors that generate all vertebrate adult blood lineages. Recent analyses have highlighted the importance of somite-derived signaling factors in regulating HSC specification and emergence from dorsal aorta hemogenic endothelium. However, these factors remain largely uncharacterized. We provide evidence that the vitamin A derivative retinoic acid (RA) functions as an essential regulator of zebrafish HSC formation. Temporal analyses indicate that RA is required for HSC gene expression prior to dorsal aorta formation, at a time when the predominant RA synthesis enzyme, *aldh1a2*, is strongly expressed within the paraxial mesoderm and somites. Previous research implicated the Cxcl12 chemokine and Notch signaling pathways in HSC formation. Consequently, to understand how RA regulates HSC gene expression, we surveyed the expression of components of these pathways in RA-depleted zebrafish embryos. During somitogenesis, RA-depleted embryos exhibit altered expression of *jam1a* and *jam2a*, which potentiate Notch signaling within nascent endothelial cells. RA-depleted embryos also exhibit a severe reduction in the expression of *cxcr4a*, the predominant Cxcl12b receptor. Furthermore, pharmacological inhibitors of RA synthesis and Cxcr4 signaling act in concert to reduce HSC formation. Our analyses demonstrate that somite-derived RA functions to regulate components of the Notch and Cxcl12 chemokine signaling pathways during HSC formation.

## Introduction

All adult vertebrate hematopoietic lineages arise from a common multipotent progenitor, the hematopoietic stem cell (HSC). This definitive hematopoietic cell type is able to self renew, differentiate into all major blood lineages, and maintain adult hematopoiesis for life. HSC transplants are used to treat a spectrum of disease ranging from congenital blood disorders to acute leukemia. Unfortunately, these cells are present in restrictive quantities, and recent *ex vivo* methods for expanding human HSCs for clinical therapies have achieved limited success. Identifying the molecular pathways that regulate HSC formation is therefore a major goal of both basic and clinical biology.

The HSC arises intraembryonically, in an anatomically distinct site from primitive hematopoietic cells [1, 2]. HSCs emerge from mesoderm-derived hemogenic endothelium, in close association with the dorsal aorta [2, 3]. In mice, this region is termed the aorta-gonad-mesonephros (AGM). Following their emergence, mammalian HSCs then migrate to the fetal liver and spleen before becoming established in the bone marrow [4, 5]. Although much is known about the cellular and functional properties of vertebrate HSCs, the genetic regulatory mechanisms that govern HSC induction from the AGM, expansion, and homeostasis remain incompletely understood. One candidate regulator of HSC formation is the vitamin A derivative retinoic acid (RA).

RA is an extremely potent diffusible morphogen. Consequently, its levels are tightly regulated within the developing embryo. The *aldehyde dehydrogenase 1a* (*aldh1a/Raldh*) genes encode the rate-limiting enzymes in RA synthesis, and high levels of RA occur in or near tissues that express them [6–8]. Mouse *Aldh1a2* (*Raldh2*) mutants recapitulate phenotypes associated with vitamin A deficiency, suggesting that Aldh1a2 is the rate-limiting source of RA in the vertebrate embryo [7, 8]. Once synthesized, RA binds nuclear retinoic acid receptor and retinoid X receptor heterodimers to activate target gene transcription [9–11].

RA has been shown to enhance the short and long-term repopulating activity of HSCs in suspension culture and serial transplantation assays [12, 13]. Conversely, treatment of HSCs with the RAR antagonist AGN 193109 reduces HSC repopulating activity [13], implicating RA signaling in HSC maintenance. *In vivo* evidence for the role of RA signaling in definitive hematopoiesis has emerged from analyses of *Aldh1a2*-mutant mice. These mice exhibit decreased numbers of yolk sac hemogenic endothelial cells, and a corresponding loss of multipotent blood progenitors that give rise to myeloid and erythroid lineages [14]. At embryonic day 8.0, *Aldh1a2*-mutants exhibit normal endothelial cell-specific gene expression, and normal circulation [14, 15]. These data suggest that RA signaling is not required for general endothelial cell formation, but rather for vascular endothelial cells to adopt a hemogenic fate. Support for this hypothesis comes from recent analyses of mice with a conditional deletion of *Aldh1a2* in VE-cadherin-positive endothelial cells [16], as AGM-derived endothelial cells isolated from these mice fail to contribute to the peripheral blood of recipients following transplant. Notably, *Aldh1a2*-mutant mice die of severe vascular defects by embryonic day 10.5 [7], prior to HSC emergence. This early embryonic lethality makes mice a challenging model in which to examine the native developmental functions of RA in definitive hematopoiesis.

Zebrafish have recently become one of the most powerful model organisms with which to study embryonic hematopoieisis. Unlike mice, Aldh1a2-depleted zebrafish survive up to five days post fertilization (dpf) [17]. Zebrafish HSC emergence occurs by 30 hours post fertilization (hpf), making zebrafish an ideal model to study the role of RA in definitive hematopoiesis. In the present study, we provide evidence that RA is an essential regulator of zebrafish HSC specification. We demonstrate that RA regulates the formation of HSCs prior to dorsal aorta hemogenic endothelial cell formation, at a time when *aldh1a2* is expressed in the paraxial mesoderm and somites. Recent evidence suggests that these mesodermal derivatives contribute to HSC formation in a Notch and Cxcl12 chemokine-dependent fashion [18–25]. We therefore sought to determine if and how these two signaling pathways are regulated by RA signaling. To do this, we conducted a comprehensive survey of Notch and Cxcl12 pathway component gene expression in RA-depleted embryos. We find that RA-depleted embryos display altered expression of the junctional adhesion molecules *jam1a* and *jam2a*, which enhance Notch signaling in pre-hematopoietic endothelial cells [26]. *cxcl12b* and its receptor *cxcr4a* are initially expressed within the somites, and later within the dorsal aorta. We find that somitic *cxcr4a* expression is strongly downregulated in RA-depleted embryos. Our work reveals a novel, early role for RA in definitive hematopoiesis and suggests that RA may regulate HSC formation by modulating the expression of Notch and Cxcl12b chemokine signaling pathway components.

## Materials and Methods

### Animal care, fish lines, and morpholino injection

Care of adult and embryonic zebrafish was conducted according to standard protocols [27], in accordance with Canadian Council for Animal Care (CCAC) guidelines. This study was approved by the University of Alberta Animal Care and Use Committee for Biosciences (protocol AUP00000082). Embryos were grown at room temperature (RT), 25.5°C, 28.5°C, or 33°C in embryo media (EM) and staged according to standardized morphological criteria [28]. EM was supplemented with 0.003%– 0.006% 1-phenyl 2-thiourea (PTU) (Sigma), to prevent pigment formation in post-24 hours post fertilization (hpf) embryos.

Unless noted, AB strain zebrafish were used for all experiments. Transgenic fish lines used in experiments include *Tg*(*gata1*:*DsRed*)^sd2Tg^ [29], and *Tg*(*kdrl*:*GFP*)^la116Tg^ [30]. Aldehyde dehydrogenase 1 family, member A2 (Aldh1a2)-depleted embryos were generated by injecting one-cell AB embryos with 5 ng of translation-blocking *aldh1a2* morpholino oligonucleotide (MO; Gene Tools); GCAGTTCAACTTCACTGGAGGTCAT, as previously described [17].

### Pharmacological treatments

A 10 μM solution of the Cxcr4 receptor antagonist AMD3100 (EMD Millipore) in EM, was used to inhibit Cxcr4 chemokine signaling [25]. Embryos were treated with AMD3100 from 4 hpf to 24 hpf. All other compounds were dissolved in Dimethyl sulfoxide (DMSO), and diluted to a working concentration in EM. Equivalent solutions of DMSO/EM were used as solvent controls. A 1 μM, 2.5 μM or 5 μM solution of Diethylaminobenzaldehyde (DEAB; Sigma) was used to inhibit retinoic acid (RA) synthesis by aldehyde dehydrogenase enzymes [31, 32]. Embryos were treated with DEAB from 4 hpf onward. A 1 nM solution of all-trans RA (Sigma) was applied to live, dechorionated embryos at various stages to activate retinoic acid signaling. All embryos were then grown at 28.5°C in the dark, and were assessed for phenotypes, washed into EM, or fixed in 4% PFA/PBS overnight at 4°C.

### mRNA *in situ* hybridization and imaging

Examination of gene expression by whole-mount *in situ* hybridization was performed essentially as previously described [33–37]. Prior to mRNA in situ hybridization analyses, embryos were fixed in 4% paraformaldehyde (PFA)/phosphate-buffered saline (PBS) overnight at 4°C or 4–5 hours at RT with gentle agitation on a rotating platform. Embryos were permeabilized in 10 μg/ml proteinase K for 10 seconds (10–12 hpf embryos), 30 seconds (14–17 hpf embryos), 3 minutes (24–32 hpf embryos), or 1 hour (3–4 days post fertilization embryos) at RT.

Following *in situ* hybridization, embryos were manually deyolked, and cleared in 30%, 50%, and 70% glycerol/PBS. Mounted *in situ* hybridized embryos and live *Tg*(*gata1*:*DsRed*)^sd2Tg^ embryos were photographed using a Zeiss AxioImager Z1 compound microscope with an Axiocam HR digital camera. Mounted *Tg*(*kdrl*:*GFP*)^la116Tg^ embryos were photographed using a Zeiss LSM510 confocal microscope. Whole embryos were photographed using an Olympus stereoscope with a QImaging micropublisher camera. Images were assembled in ImageJ or Zen (Zeiss), and figures were assembled in Photoshop (Adobe).

### Real-time quantitative PCR (qPCR)

mRNA was extracted from dissected (head and tail removed) embryos using RNAqueous-4PCR (Ambion) according to the manufacturer’s specifications, then treated with DNase I (Ambion), 19 μl of diethylpyrocarbonate-treated water, and 10 μl of 10X DNase I Buffer (Ambion) for 30 min at 37°C to remove DNA. Extracted mRNA was purified using the RNeasy Mini Kit (Qiagen) according to the manufacturer’s specifications. RNA quantity and quality was assessed by spectrophotometry. First-strand cDNA synthesis was performed using the AffinityScript QPCR cDNA Synthesis Kit (Agilent), with random primers, according to the manufacturer’s specifications.

qPCR analysis of cDNA was performed using the Brilliant II SYBR Green QPCR Master Mix (Agilent) and the Rotor-Gene Q System (Qiagen). All cDNA samples were run in replicates of 6, and each experiment was repeated three times. The PCR cycle conditions were 95°C for 10 min (initial denaturation), then 40 cycles of 95°C for 30 s (denaturation), 55°C for 1 min (annealing), and 72°C for 30 s (extension). Fluorescence readings were taken after the 55°C annealing step. The Ct value data were analyzed using the comparative Ct method (2^-ΔΔCt^ method) [38], using *eukaryotic translation elongation factor 1 alpha 1a* (*ef1a*) as an endogenous control. Previously published qPCR primer sequences are *cxcr4a*-F, TGGCTTATTACGAACACATCG; *cxcr4a*-R GAGCCGAATTCAGAGCTGTT [39]; *ef1a*-F, CCTTCGTCCCAATTTCAGG; *ef1a*-R, CCTTGAACCAGCCCATGT [34]. Intron-spanning *her9* (*her9*-F, GAATGCCAGCGAGCATAG; *her9*-R, GCTTGACTGTCATCTCCA G) qPCR primers were selected from the Universal Probe Library Assay Design Center for Zebrafish (Roche), Prior to real-time qPCR analysis, these primer sets were validated as follows: An amplification plot was produced from a standard cDNA two-fold dilution series. This plot was used to generate a linear regression curve. The validated *her9* primer sets produced a linear regression slope of -3.3 ± 0.1 (within 0.1 of the *ef1a* primer set), with a coefficient of determination (*R*^2^) of 0.99.

### Morphometric Analysis

For morphometric analysis, data from three independent replicates with separate cohorts of zebrafish embryos were analyzed for the main effects of treatment. All measurements were performed using ImageJ software. Width of somitic *jam2a* expression was measured as a fraction of the length of the domain of expression along the medio-lateral axis (denoted *y*), divided by length of the domain of expression along the anterior-posterior axis (denoted *x*) of the eighth *jam2a*-expressing somite on the right side of the embryo (fraction *y*/*x*). Notably, the length of the domain of *jam2a* expression along the anterior-posterior axis (x) of the eighth *jam2a*-expressing somite did not significantly differ in control (DMSO) versus DEAB-treated embryos (*P* ≥ 0.2624).

### Statistical analyses

For *in situ* hybridization experiments, and analyses of circulation in *Tg*(*gata1*:*DsRed*)^sd2Tg^ embryos, data from two to three independent replicates with separate cohorts of zebrafish embryos were analyzed for the main effects of treatment. Homogeneity across replicates was determined using the *G*-test of independence, and homogenous datasets (heterogeneity *G*-value ≥ 0.05) were combined for statistical analysis. Heterogeneous replicate datasets were analyzed separately, and combined when statistical analyses yielded identical results for each replicate dataset. Significant differences among treatments were determined using two-tailed Fisher’s exact tests on cumulative raw counts, with Bonferroni correction applied to multiple comparisons (alpha = 0.05). For DEAB and AMD3100 treatment experiments, significant differences in *cmyb*-expressing dorsal aorta cell counts among treatments were determined using unpaired t-tests, with Bonferonni correction applied to multiple comparisons (alpha = 0.05). For qPCR analyses and morphometric analysis, significant differences among treatments were determined using unpaired t-tests (alpha = 0.05).

For analyses of dorsal aorta morphology in *Tg*(*kdrl*:*GFP*)^la116Tg^ embryos, data from three independent replicates with separate cohorts of zebrafish embryos were analyzed for the main effects of treatment. Significant differences among treatments and phenotypes were determined by two-way ANOVA with Bonferroni’s post-test (alpha = 0.05).

## Results

### Retinoic acid regulates hematopoietic stem cell formation

**Fig 1:**
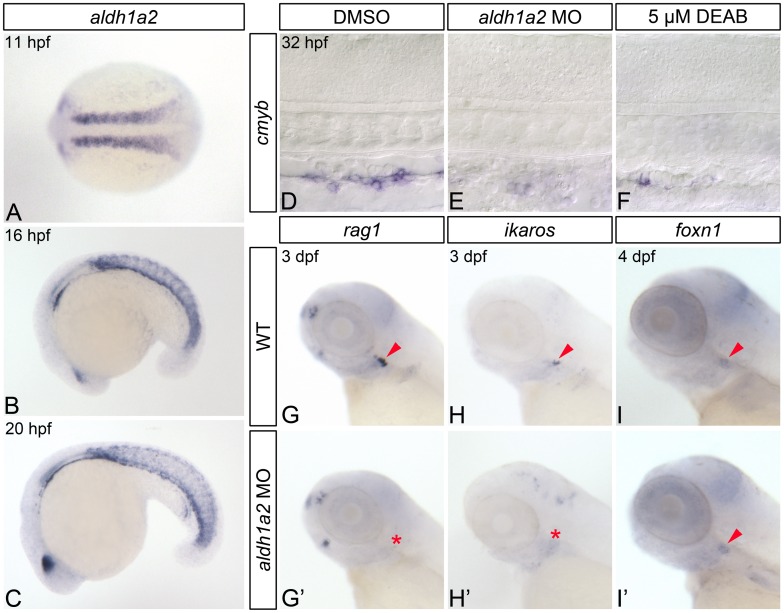
RA-deficient embryos demonstrate impaired HSC formation. (A-C) *In situ* hybridization analyses of *aldh1a2* gene expression in wild type (WT) embryos. (A) Expression within the somites at 11 hpf, shown in dorsal view with anterior to the left. Somitic expression persists in 16 hpf (B) and 20 hpf (C) embryos, shown in lateral view with anterior to the left. (D-F) Representative flat-mounted embryos following *in situ* hybridization analysis of *cmyb* gene expression at 32 hpf. Lateral view of gene expression in the dorsal aorta region of the trunk is shown with anterior to the left. Compared to DMSO-treated controls (D) *aldh1a2*-morphants (E), and 5 μM DEAB-treated embryos (F) exhibit nearly abolished *cmyb* expression. (G-H’) *In situ* hybridization analyses of common lymphoid progenitor gene expression in 3 dpf embryos. Lateral view of gene expression in the head is shown with anterior to the left. Arrowheads and asterisks indicate thymus. Compared to WT embryos (G, H), *aldh1a2*-morphants exhibit nearly abolished thymic *rag1* (G’) and *ikaros* (H’) expression. (I, I’) Representative embryos following *in situ* hybridization analysis of *foxn1* thymic epithelial cell gene expression in 4 dpf embryos. Lateral view of gene expression in the head is shown with anterior to the left. Arrowheads indicate thymus. WT embryos (I) and *aldh1a2*-morphants (I’) exhibit similar thymic *foxn1* expression levels.

Of the known *aldh1a* genes expressed in early zebrafish development, only *aldh1a2* is expressed in pre-hematopoietic posterior mesoderm (Fig 1A–1C; [40–42]). Consequently, to determine if RA regulates zebrafish HSC formation, we generated RA-deficient embryos by injecting embryos with *aldh1a2* morpholino (hereafter referred to as *aldh1a2*-morphants) [41, 43], or by treating them with Diethylaminobenzaldehyde (DEAB), a competitive inhibitor of aldehyde dehydrogenases including Aldh1a2 [31, 32]. Published analyses indicate that *aldh1a2*-morphants and DEAB treatment accurately phenocopy *aldh1a2* (*nls*^*i26*^)-mutants [31, 41]. Zebrafish HSCs first emerge from dorsal aorta hemogenic endothelium, a region analogous to the mammalian aorta-gonad-mesonephros, at 30 hours post fertilization (hpf) [44–47]. These cells express *cmyb*, a transcription factor essential for HSC emergence [47]. As shown by *in situ* hybridization, both *aldh1a2*-morphants and DEAB-treated embryos display severely reduced dorsal aorta *cmyb*-expression at 32 hpf (Fig 1D–1F; S1 Table).

Following their emergence, zebrafish HSCs migrate posteriorly to the caudal hematopoietic tissues, before becoming established in the thymus by 3 dpf [48, 49], where they differentiate to form *rag1*- and *ikaros*-expressing lymphoid progenitors [48, 49]. Subsequently, in order to further determine if HSCs are specified in RA-deficient zebrafish embryos, we examined their *rag1* and *ikaros* expression. *aldh1a2*-morphant embryos completely lack thymic *rag1* and *ikaros* expression at 3 dpf, as shown by *in situ* hybridization (Fig 1G and 1H’; S2 Table).

Thymic epithelial cells support lymphoid progenitor development and maturation. These cells differentiate from the thymus primordium, which is derived from the third pharyngeal endodermal pouch in zebrafish [50]. As perturbations in RA signaling have been shown to produce defects in endodermal pouch morphogenesis [51], we wanted to verify that the thymic epithelium of RA-deficient embryos is correctly specified. We therefore examined the expression of the thymic epithelial cell marker *foxn1*, and find that it is expressed at wild type levels in 4 dpf *aldh1a2*-morphant embryos (Fig 1I and 1I’; S2 Table). Combined, our data suggest that RA is required for the proper specification of zebrafish HSCs and their thymocyte progeny.

**Fig 2:**
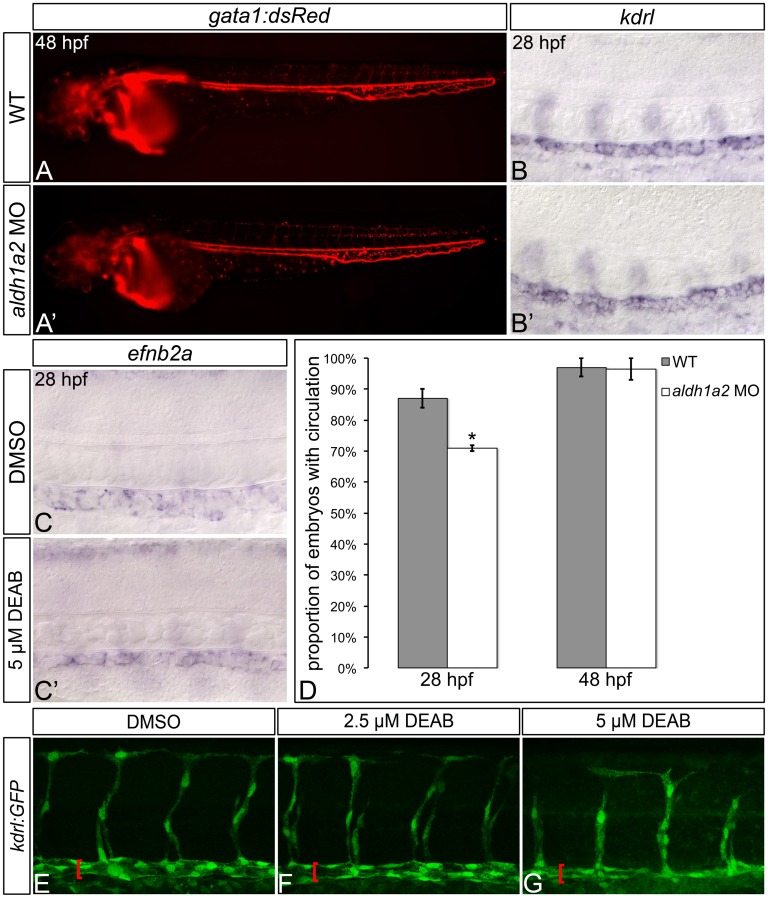
RA-deficient embryos exhibit relatively normal gross embryonic vasculogenesis. (A, A’) Lateral view of live 48 hpf *Tg*(*gata1*:*DsRed*)^sd2Tg^ embryos with anterior to the left. Compared to wild type (WT) embryos (A), *aldh1a2*-morphants (A’) display visible circulating blood cells, and an intact dorsal aorta and posterior cardinal vein. (B-C’) Representative embryos following *in situ* hybridization analysis of *kdrl* vasculature marker gene expression (B, B’) or *efnb2a* arterial marker gene expression (C, C’) in 28 hpf embryos. Lateral view of gene expression in the dorsal aorta region of the trunk is shown in flat-mount embryos, with anterior to the left. Compared to WT embryos (B), *aldh1a2-*morphants (B’) exhibit normal dorsal aorta *kdrl* gene expression. Compared to DMSO-treated controls (C), embryos treated with 5 μM DEAB (C’) exhibit normal levels, but a reduced domain of dorsal aorta *efnb2a* gene expression. (D) Graph demonstrating the mean proportion of WT or *aldh1a2*-morphant embryos with intact circulation at 28 hpf and 48 hpf. Error bars represent standard error. *Indicates statistically significant difference compared to WT (*P* = 0.0196). See text for statistical tests. (E-G) Lateral view of dorsal aorta region of the trunk is shown in representative flat-mount *Tg*(*kdrl*:*GFP*)^la116Tg^ 28 hpf embryos, with anterior to the left. Brackets indicate dorsal aorta. Compared to DMSO-treated controls (E), 2.5 μM DEAB-treated embryos (F) exhibit normal dorsal aorta morphology, while 5 μM DEAB-treated embryos (G) exhibit thinning of the dorsal aorta.

As HSC formation is also dependent upon intact blood flow [52], and HSCs originate from dorsal aorta hemogenic endothelium [44–46], we next wanted to determine if the hematopoietic defects that we observe in RA-deficient embryos are due to aberrant vasculogenesis. To do this, we first visualized circulating primitive erythrocytes in wild type and *aldh1a2*-morphant embryos. The majority of *aldh1a2-*morphants (71% ± 1.0%) exhibit circulating blood cells by 28 hpf (Fig 2D; S3 Table). However, this represents a significant reduction when compared to wild type embryos (87% ± 3.0%, *P* = 0.0196; Fig 2D; S3 Table). Examination of live 48 hpf, *aldh1a2*-morphant *Tg*(*gata1*:*DsRed*)^sd2Tg^ [29] embryos reveals beating hearts, intact circulation, and a functional dorsal aorta (Fig 2A and 2A’). At 48 hpf, the proportion of *aldh1a2*-morphants with intact circulation (97% ± 3.5%) is not statistically different from that of wild type embryos (97% ± 3.0%, *P* = 0.5633; Fig 2D; S3 Table). Combined, these data suggest that some *aldh1a2*-morphants experience a mild delay in the formation of their mature circulatory system.

To determine if the hematopoietic defects of RA-deficient embryos are attributable to alterations in dorsal aorta morphogenesis and patterning, we examined dorsal aorta morphology in *Tg*(*kdrl*:*GFP*)^la116Tg^ transgenic zebrafish embryos [30] at 28 hpf, following the onset of circulation (Fig 2E–2G; S4 Table). Compared to control embryos (Fig 2E; 100% ± 0%), the majority of embryos treated with 2.5 μM DEAB exhibit grossly normal vasculature (Fig 2F; 69% ± 20%; *P* ≥ 0.05). Conversely, only 19% ± 13% of embryos treated with a higher 5 μM dose of DEAB exhibit wild type dorsal aorta morphology (*P* < 0.05; Fig 2G). As shown by *in situ* hybridization, RA-deficient embryos exhibit wild type *kdrl* vasculature marker expression and wild type levels of *efnb2a* arterial marker gene expression at 28 hpf (Fig 2B and 2C’; S5 Table). Our combined data therefore suggest that RA does not regulate vascular or arterial gene expression. Our data also suggest that low doses of DEAB (2.5 μM) can be used to block RA synthesis without causing gross maldevelopment of the embryonic vasculature. To avoid generating confounding hematopoietic phenotypes that result from impaired circulation, we used 2.5 μM DEAB or *aldh1a2* morpholino to deplete RA when assessing circulation-stage (26–32 hpf) embryos in all subsequent experiments.

### RA is dispensable for zebrafish dorsal aorta Notch1 signaling

Previous studies have revealed an essential role for the Notch signaling pathway in regulating vertebrate HSC development [18, 19, 24, 53–55]. Binding of the transmembrane Notch receptor to its Delta or Jagged transmembrane ligand on a neighboring cell induces a conformational change in Notch that renders it susceptible to cleavage by γ-secretase. This cleavage event releases the Notch intracellular domain (NICD), permitting it to enter the nucleus where it acts as a transcriptional activator [56, 57]. The basic helix-loop-helix transcription factors *Hairy and enhancer of split* (*Hes*) are transcriptional targets of the Notch signaling pathway, and serve to mediate the majority of Notch function [58].

**Fig 3:**
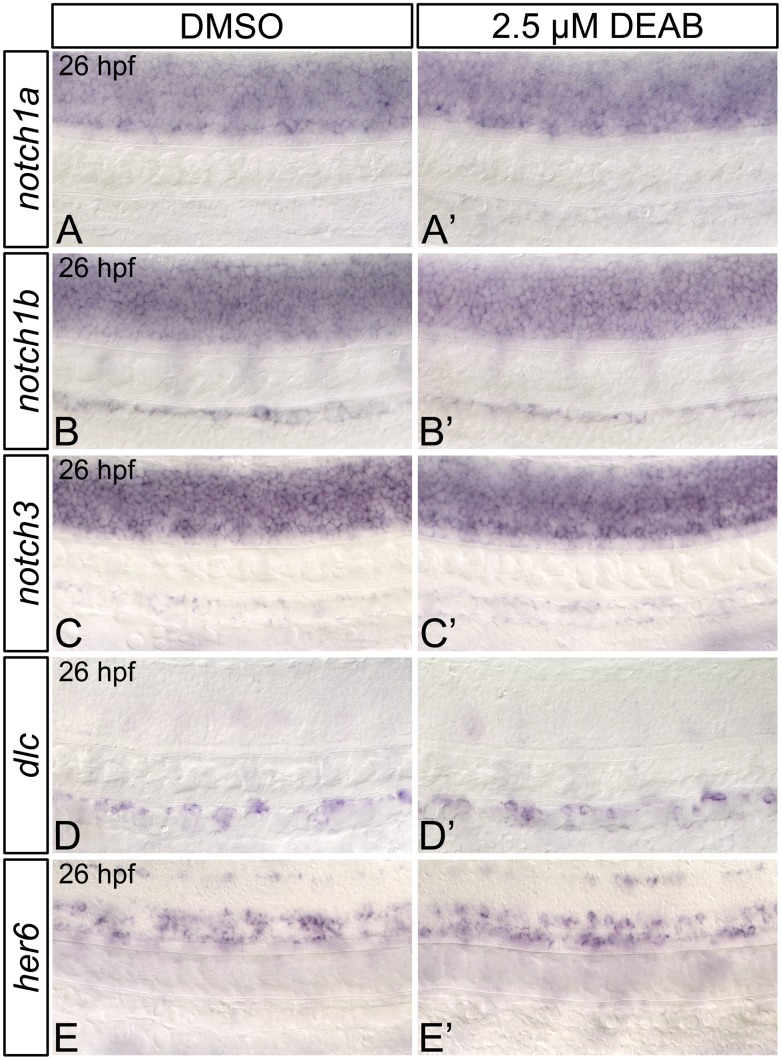
RA-deficient embryos demonstrate normal dorsal aorta *notch* and Notch1-target gene expression. Representative flat-mount 26 hpf embryos following *in situ* hybridization analyses. Lateral view of gene expression in the dorsal aorta region of the trunk is shown with anterior to the left. Compared to DMSO-treated controls (A, B), embryos treated with 2.5 μM DEAB exhibit normal *notch1a* (A’), and *notch1b* (B’) gene expression within the trunk and dorsal aorta. *notch3* is expressed at normal levels in the dorsal aorta (C, C’), but is mildly upregulated in the somites of 2.5 μM DEAB-treated versus DMSO-treated control embryos (data not shown). Compared to DMSO-treated controls (D, E) embryos treated with 2.5 μM DEAB exhibit normal gene expression levels of the Notch1-signaling pathway transcriptional targets *dlc* (D’) and *her6* (E’).

Previous research in both mouse and zebrafish has established a cell-autonomous function for Notch signaling in HSC specification, whereby Notch1-expressing cells within the dorsal aorta are instructed by adjacent cells to form HSCs [19–24]. The yolk sac endothelial cells of *Aldh1a2*-mutant mice exhibit downregulated *Notch1* and Notch1-target gene (*Hes1*) expression [59], implicating RA as a potential modulator of Notch signaling. We therefore wanted to determine if hematopoietic defects that we observe in RA-deficient zebrafish are the result of impaired Notch1 signaling. To do this, we first examined the expression of dorsal aorta Notch signaling pathway components and their downstream transcriptional targets in RA-deficient embryos. Zebrafish possess four Notch receptors: Notch1a, Notch1b, Notch2, and Notch3. Of these, only Notch2 is completely dispensable for HSC formation [20–22]. *notch1a*, *notch1b*, and *notch3* are initially expressed within the somitic mesoderm, with their domain of expression expanding to include nascent endothelial cells and the dorsal aorta [21, 53, 60]. As shown through *in situ* hybridization, RA-deficient embryos exhibit wild type expression of *notch1a*, and *notch1b*, while the somitic expression of *notch3* is mildly increased at 26 hpf (Fig 3A–3C’; S6 Table). Our combined data suggest that RA is dispensable for dorsal aorta Notch receptor expression in zebrafish.

Previous research has shown that both global NICD induction after 20 hpf, and vascular (but not somite)-specific induction of the NICD rescues the HSC gene expression defects of *notch1a-* and *notch1b*-morphant zebrafish embryos [21]. Conversely, global or somite-specific NICD induction at 14 hpf, but not 20 hpf, rescues HSC formation in *notch3*-morphant embryos [21]. As the dorsal aorta begins to form at 20 hpf [61], these combined data suggest that the definitive hematopoietic roles of zebrafish Notch1a/b and murine Notch1 are likely functionally conserved. These data also suggest that there is a distinct temporal and spatial requirement for Notch3 in zebrafish hematopoiesis, which occurs prior to formation of the dorsal aorta. Consequently, to further determine if RA regulates zebrafish dorsal aorta Notch signaling, we next examined the expression of transcriptional targets of the Notch1-signaling pathway in RA-deficient embryos (Fig 3D and 3E’; S6 Table). Expression of the Notch ligand *deltaC* (*dlc*) is strongly reduced in the dorsal aorta of *notch1a-* and *notch1b*-morphant zebrafish embryos [21]. We therefore examined its expression in RA-deficient embryos. *dlc* is expressed at wild type levels in 26 hpf DEAB-treated embryos (Fig 3D and 3D’; S6 Table). We also examined the expression of *her6* (the zebrafish orthologue of mammalian *Hes1* [62, 63]), finding that is also expressed at wild type levels in 26 hpf *aldh1a2*-morphant embryos (Fig 3E and 3E’; S6 Table). These data suggest that, unlike its mammalian orthologue *Hes1*, zebrafish *her6* is not RA-responsive. These combined data also suggest that RA does not regulate the Notch1-signaling pathway in zebrafish.

### RA signaling regulates HSC formation prior to 19 hpf

**Fig 4:**
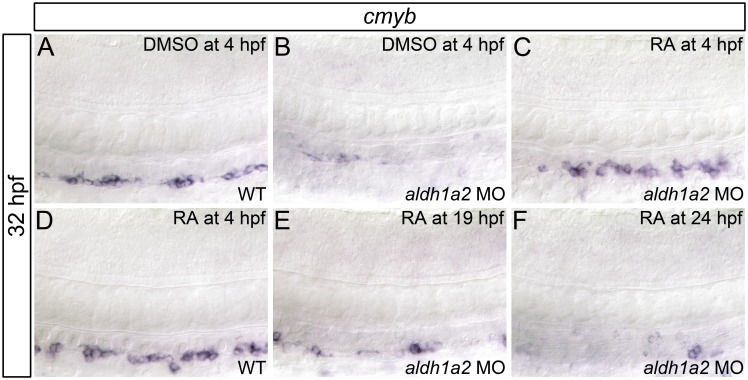
RA is required prior to 19 hpf for HSC formation. Shown are representative flat-mount embryos following *in situ* hybridization analyses of *cmyb* gene expression in wild type (WT; A, D) or *aldh1a2*-morphant (B, C, E, F) 32 hpf embryos treated with DMSO (A, B) or 1 nM RA (C-F) at indicated time points. Lateral view of gene expression in the dorsal aorta region of the trunk is shown with anterior to the left. Compared to WT embryos (A), embryos treated with 1 nM RA (D) exhibit normal *cmyb* expression (two-tailed *P* = 1.000). *aldh1a2*-morphants (B) exhibit nearly abolished *cmyb* expression compared to WT embryos (two-tailed *P* = 0.01). *cmyb* expression is significantly restored in *aldh1a2*-morphant embryos treated with 1 nM RA at 4 hpf (C; two-tailed *P* = 1.000 compared to WT). *cmyb* expression is not significantly restored in *aldh1a2*-morphants treated with 1 nM RA at 19 hpf (E; two-tailed *P* < 0.0005) or 24 hpf (F; two-tailed *P* < 0.0005). See text for statistical tests.

Our analyses indicate that the hematopoietic defects of RA-deficient embryos are not due to impaired dorsal aorta Notch signaling. Consequently, to gain a better understanding of how RA regulates zebrafish definitive hematopoiesis, we next wanted to elucidate the temporal requirement for RA signaling in HSC formation. To accomplish this, we treated *aldh1a2*-morphant embryos with RA at different time points, and examined their *cmyb* HSC gene expression at 32 hpf through *in situ* hybridization (Fig 4, S7 Table). We demonstrate that RA treatment beginning at 4 hpf rescues dorsal aorta *cmyb* gene expression in *aldh1a2*-morphant embryos (Fig 4C). Conversely, RA treatment beginning at 19 hpf (Fig 4E) or 24 hpf (Fig 4F) fails to rescue *cmyb* expression in *aldh1a2*-morphant embryos. Combined, these data suggest that RA is required prior to 19 hpf to specify HSCs. Notably, *aldh1a2* is expressed in the paraxial mesoderm and somites during this period, and the dorsal aorta has not yet formed [61].

### RA does not positively regulate Wnt16 –Notch3 signaling within the somites

**Fig 5:**
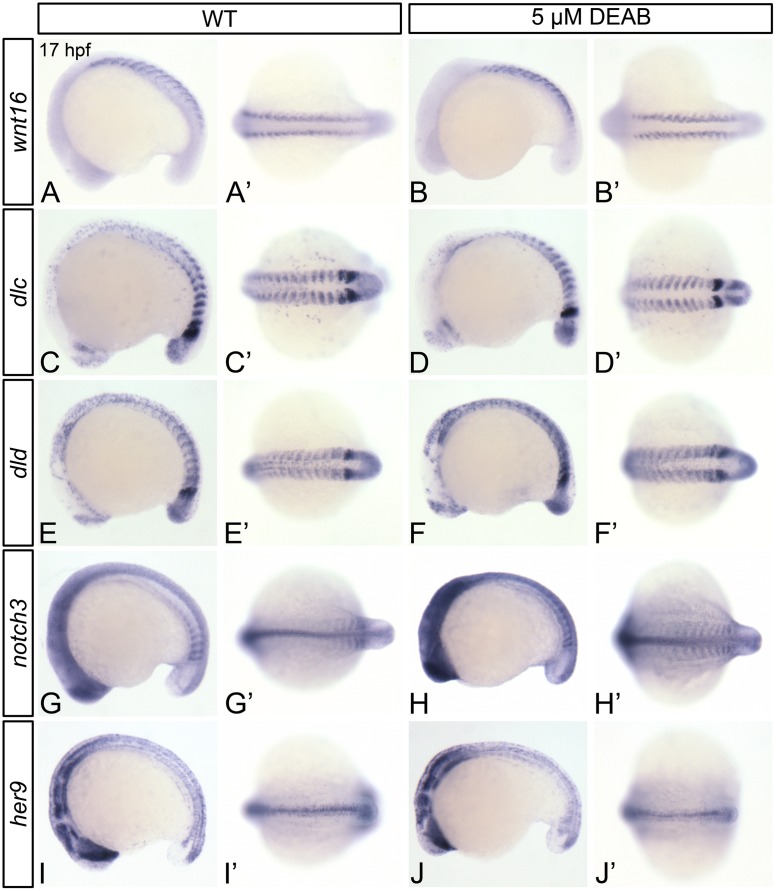
RA does not regulate the somitic expression of Wnt16-Notch3 signaling pathway components. Shown are representative 17 hpf embryos following *in situ* hybridization analyses (A-J’). Lateral view (A-J) or dorsal view (A’-J’) of gene expression is shown with anterior oriented to the left. A’-J’ represent different views of the embryos shown in A-J. Compared to DMSO-treated controls (A, A’, C, C’, E, E’, G, G’), DEAB-treated embryos exhibit normal somitic expression levels of *wnt16* (B, B’), and *dlc* (D, D’), mildly increased *dld* expression (F, F’), and increased *notch3* somitic gene expression (H, H’). DEAB-treated embryos also exhibit normal expression levels of the Notch3 signaling pathway transcriptional target *her9* (J, J’), when compared to DMSO-treated controls (I, I’). (K) Quantitative real-time PCR analysis of *her9* expression in 17 hpf DMSO-treated controls and embryos treated with 5 μM DEAB. Shown is the relative quantity of *her9* expression. Samples were normalized to *ef1a* and DMSO-treated was set to 1. Error bars indicate standard error of the mean. *Indicates the difference compared to control is significant by Student *t* test, *P* = 0.0198.

The Wingless-type MMTV integration site family, member 16 (Wnt16) participates in a non-canonical Wnt signaling pathway [18]. Its depletion causes defects in Notch signaling and HSC formation [18]. Given that RA and Wnt16 are required for HSC formation prior to 19 hpf, and *aldh1a2* and *wnt16* are both expressed in the paraxial mesoderm and somites at this time [18], we hypothesized that perturbations in Wnt16 or its downstream effectors may be responsible for the hematopoietic defects that we observe in RA-depleted embryos. We demonstrate that *wnt16* is expressed at wild type levels DEAB-treated embryos at 17 hpf (Fig 5A and 5B’; S8 Table). In addition to hematopoietic defects, Wnt16-depleted embryos exhibit reduced somitic expression of the Notch ligands *dlc* and *dld*, and *dlc/dld* overexpression rescues HSC gene expression in *wnt16*-morphants [18]. In comparison to wild type embryos, DEAB-treated embryos exhibit normal levels of somitic *dlc* expression (Fig 5C and 5D’; S8 Table), and mildly upregulated *dld* expression (Fig 5E and 5F’; S8 Table). Dlc/Dld and Notch3 proteins cooperate as regulators of HSC formation, as partial loss of Dlc and Notch3, or Dld and Notch3 produces greater HSC gene expression defects than partial loss of Dlc, Dld, or Notch3 alone [21]. When compared to wild type embryos (Fig 5G and 5G’), DEAB-treated embryos exhibit increased somitic *notch3* expression at 17 hpf (Fig 5H and 5H’; S8 Table). *her9* is partially downregulated in both *notch1a*-mutant and *notch3*-morphant zebrafish embryos [60, 64]. Like *notch3*, *her9* expression is upregulated in 17 hpf DEAB-treated embryos, as shown by qPCR (Fig 5K). However, this upregulation in expression is not observable by *in situ* hybridization (Fig 5I and 5J’; S8 Table). Taken together, our data provide evidence that RA negatively regulates Notch3-mediated signal transduction, without altering somitic Wnt16.

### RA regulates *jam1a* and *jam2a* expression

The dorsal aorta forms from angioblasts that arise from bilateral stripes of posterior lateral-plate mesoderm. These angioblasts migrate medially and aggregate [61]. Recent evidence suggests that the junctional adhesion molecules Jam1a and Jam2a physically interact, and are required for zebrafish HSC formation [26]. *jam1a* is expressed within angioblasts that migrate across j*am2a*-, *dlc-*, and *dld*-expressing somites [26]. Jam1a- and Jam2a-deficient embryos exhibit impaired Notch signal transduction, and their hematopoietic defects are rescued by heat-shock induction of the NICD during angioblast migration [26]. Combined, the data generated by Kobayashi et al. (2014) suggest that Notch signal transduction in pre-hematopoietic angioblasts requires Jam-mediated intercellular contact.

**Fig 6:**
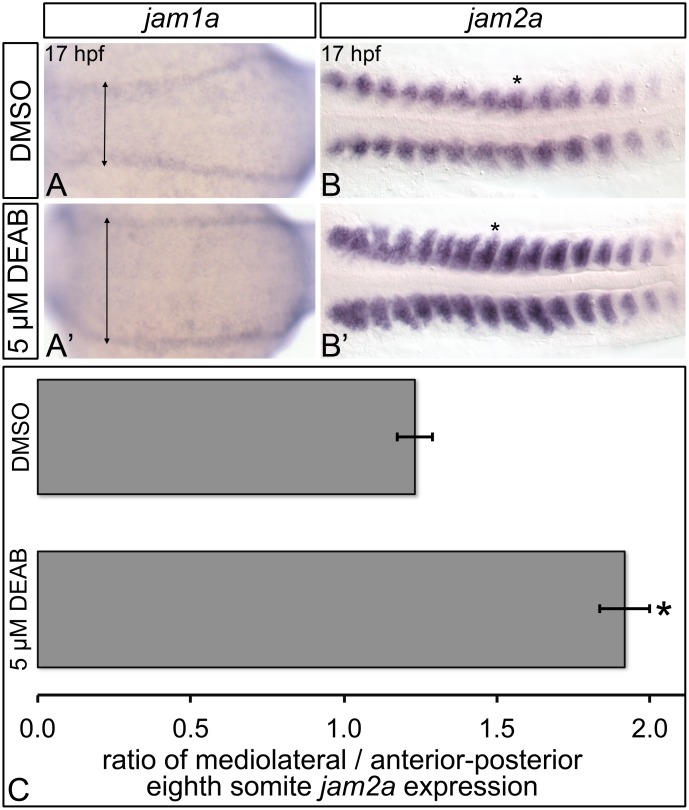
RA-deficient embryos exhibit abnormal *jam1a* and *jam2a* expression. Representative flat-mount 17 hpf embryos following *in situ* hybridization analyses. Dorsal view of gene expression is shown with anterior to the left. Compared to DMSO-treated controls (A), embryos treated with 5 μM DEAB (A’) exhibit wild type levels of *jam1a* expression, and extreme lateral positioning of the anterior-most domains of *jam1a* expression (double-headed arrows). Compared to DMSO-treated controls (B), embryos treated with 5 μM DEAB (B’) display strongly increased somitic *jam2a* expression, and lateral expansion of the *jam2a* expression domain. (C) Graph demonstrating length of the domain of *jam2a* expression along the medio-lateral axis, divided by length of the domain of expression along the anterior-posterior axis of the eighth *jam2a*-expressing somite on the right side of the embryo (see asterisk in B, B’). Error bars represent standard error *Indicates statistically significant difference in ratio compared to DMSO-treated controls (*P* < 0.0001). See text for statistical tests.

Angioblast migration occurs between 14 and 18 hpf. We demonstrate that the diffusible morphogen RA is required prior to 19 hpf for HSC formation. Furthermore, like Jam1a- and Jam2a-depleted embryos, RA-deficient embryos do not display reduced expression of *notch1a*, *notch1b*, *notch3*, *dlc*, and *dld* [26]. We therefore sought to determine if RA is an upstream regulator of *jam1a* and/or *jam2a* by examining their expression in 17 hpf control and DEAB-treated embryos through *in situ* hybridization (Fig 6; S8 Table). DEAB-treated embryos express *jam1a* at wild type levels (Fig 6A and 6A’). However, unlike in controls, the anterior-most *jam1a*-expressing posterior lateral-plate mesoderm cells do not contact the somites in DEAB-treated embryos (Fig 6A and 6A’; arrowheads). Compared to controls, 17 hpf DEAB-treated embryos display increased levels of somitic *jam2a* expression, and lateral expansion of the *jam2a* expression domain (Fig 6B, 6B’ and 6C). Combined, these data suggest that RA is required for the proper expression of *jam1a* and *jam2a* within somitogenesis stage embryos.

### RA regulates *cxcl12b* and *cxcr4a* expression

**Fig 7:**
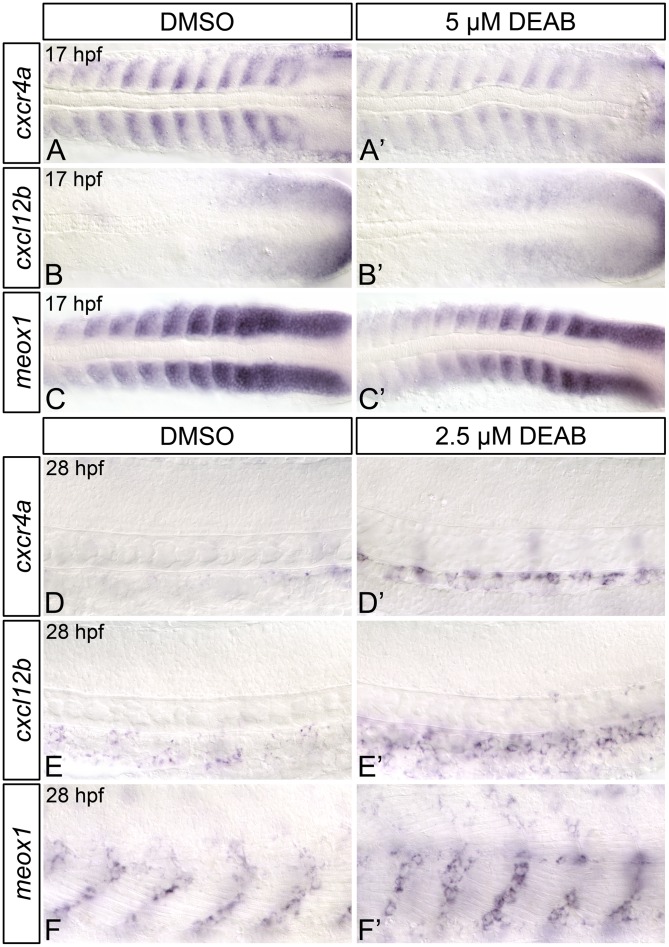
RA-deficient embryos exhibit altered Cxcl12b chemokine signaling pathway component gene expression. (A-C’) Representative flat-mount 17 hpf embryos following *in situ* hybridization analyses. Dorsal of gene expression is shown with anterior to the left. Compared to DMSO-treated controls (A), embryos treated with 5 μM DEAB (A’) exhibit strongly reduced somitic *cxcr4a* gene expression, and narrowing of the *cxcr4a* expression domain within each somite. Compared to DMSO-treated controls (B, C), embryos treated with 5 μM DEAB exhibit subtly increased levels of somitic *cxcl12b* expression (B’), and subtly decreased levels of somitic *meox1* expression (C’). (D) Quantitative real-time PCR analysis of *cxcr4a* expression in 17 hpf DMSO-treated controls and embryos treated with 5 μM DEAB. Shown is the relative quantity of *cxcr4a* expression. Samples were normalized to *ef1a* and DMSO-treated was set to 1. Error bars indicate standard error of the mean. *Indicates the difference compared to control is significant by Student *t* test, *P* < 0.0382. (E-H) Representative flat-mount 36 hpf embryos following *in situ* hybridization analyses of *cmyb* gene expression. Lateral view of gene expression in the dorsal aorta region of the trunk is shown with anterior to the left. Compared to DMSO-treated controls (E), embryos treated with 1 μM DEAB (F) or 10 μM AMD3100 (G) exhibit a small reduction *cmyb*-expressing cell numbers. Embryos treated with both 1 μM DEAB and 10 μM AMD310 (H) exhibit a more severe reduction in *cmyb*-expressing cell numbers. (I) Graph demonstrating the mean number of dorsal aorta *cmyb*-expressing cells in DMSO-treated controls, embryos treated with 1 μM DEAB, 10 μM AMD3100, or both 1 μM DEAB and 10 μM AMD310. Error bars represent standard error. *Indicates statistically significant difference compared to control (*P* ≤ 0.0144). **Indicates statistically significant difference compared to 1 μM DEAB, and 10 μM AMD3100 (*P* ≤ 0.0028). See text for statistical tests.

The CXC-motif chemokine receptor Cxcr4a and its ligand Cxcl12b regulate brain [65], coronary [66], gastrointestinal [67, 68], kidney [69], and arterial [70] vessel development, as well as lateral dorsal aorta formation [71]. Cxcl12 signaling has also been implicated in hematopoietic cell migration [72–75], engraftment [74] and hematopoietic stem cell maintenance [73, 74, 76, 77]. Recently, Nguyen et al (2014) demonstrated that Cxcl12b is required for zebrafish HSC formation, as HSC gene expression is reduced in both *cxcl12b*-morphants and embryos treated with a pharmacological inhibitor of Cxcl12 signaling from 14 to 24 hpf. Combined, these data suggest that Cxcl12b signaling within the somites contributes to zebrafish HSC formation between 14 and 24 hpf. *aldh1a2* is expressed within the somites during this period, and our data suggest that RA is required for HSC formation prior to 19 hpf. We therefore hypothesized that the HSC gene expression defects that we observe in RA-depleted embryos may be due to reduced Cxcl12 signaling, and so examined the expression of *cxcr4a* and *cxcl12b* in 17 hpf control and DEAB-treated embryos through *in situ* hybridization (Fig 7A–7C’; S9 Table). Compared to controls, 17 hpf DEAB-treated embryos exhibit narrowing of the *cxcr4a* expression domain within each somite, along with an overall strong reduction in *cxcr4a* expression (Fig 7A and 7A’; S9 Table). Conversely, *cxcl12b* expression is subtly upregulated within the somites of DEAB-treated embryos (Fig 7B and 7B’; S9 Table). We performed real-time quantitative PCR (qPCR) on 17 hpf control and DEAB-treated embryos to quantitatively measure the observed changes in *cxcr4a* expression. Consistent with the *in situ* hybridization analyses, DEAB-treated embryos exhibit a significant reduction in *cxcr4a* expression (Fig 7D) when compared to controls.

*meox1*-null (*cho*) zebrafish mutants exhibit an increase in somitic *cxcl12b* expression, and a corresponding increase in HSC number [25]. Given that we observe a subtle increase in somitic *cxcl12b* expression within RA-depleted embryos, we next wanted to determine if *meox1* expression is also affected by loss of embryonic RA. As shown by *in situ* hybridization, 17 hpf DEAB-treated embryos display a subtle decrease in somitic *meox1* expression, when compared to controls (Fig 7C and 7C’; S9 Table).

Given that RA-depleted embryos exhibit strongly decreased somitic *cxcr4a* expression, we next wanted to determine if this decrease is consistent with reduced chemokine signalling. We therefore examined if RA signalling functions in association with Cxcr4-mediated chemokine signalling to regulate HSC formation. To test this, we examined *cmyb* HSC gene expression in 36 hpf embryos treated with a suboptimal dose DEAB (1 μM) and/or a suboptimal dose of Cxcr4 chemokine receptor antagonist AMD3100 (10 μM) by *in situ* hybridization. Compared to DMSO-treated controls (Fig 7E and 7I), embryos treated with 1 μM DEAB or 10 μM AMD3100 exhibit a small reduction in *cmyb*-expressing cell numbers (Fig 7F, 7G and 7I). Embryos treated with both 1 μM DEAB and 10 μM AMD3100 exhibit a severe reduction in *cmyb*-expressing cell numbers (Fig 7H and 7I), a phenotype that resembles treatment with a higher dose of DEAB (5 μM) alone (Fig 1F). Taken together, these data support a role for somitic retinoids in regulating Cxcr4-mediated chemokine signaling during the developmental period in which RA functions to regulate HSC formation.

**Fig 8:**
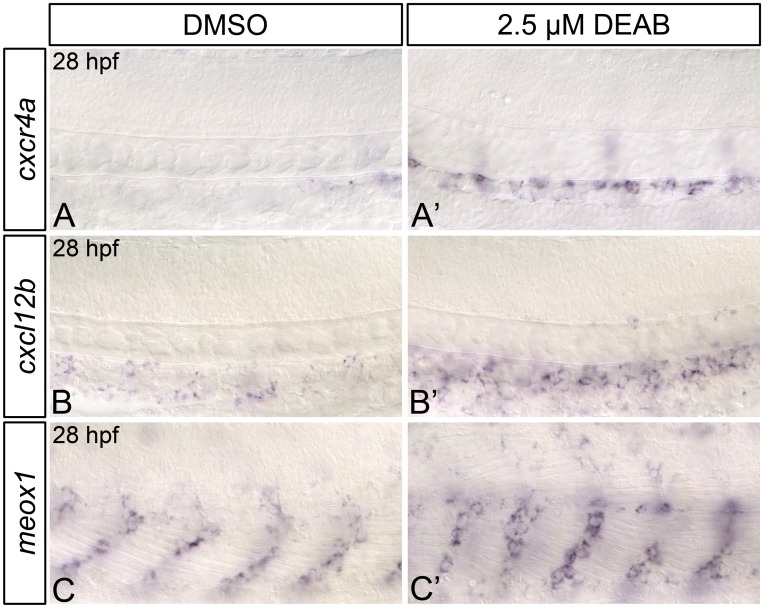
28 hpf RA-deficient embryos exhibit increased Cxcl12b chemokine signaling pathway component gene expression. (A-C’) Representative flat-mount 28 hpf embryos following *in situ* hybridization analyses. Lateral view of gene expression in the dorsal aorta region of the trunk is shown with anterior to the left. Compared to DMSO-treated controls (A, B, C), embryos treated with 2.5 μM DEAB exhibit strongly increased levels of dorsal aorta *cxcr4a* (A’), and *cxcl12b* (B’) gene expression, and upregulated somitic *meox1* expression (C’).

From 28–30 hpf, *meox1* is expressed within cells found immediately adjacent to the dorsal aorta, while *cxcr4a*, and *cxcl12b* display weak, punctate expression throughout the dorsal aorta (Fig 8A–8C; [25]). It is possible that Cxcl12b-Cxcr4a signaling within the dorsal aorta is required for HSC formation. We therefore wanted to determine if RA-depleted embryos exhibit alterations to *cxcr4a*, *cxcl12b*, and *meox1* expression at 28 hpf, just prior to HSC emergence. When compared to controls, DEAB-treated embryos exhibit a strong increase in dorsal aorta *cxcr4a* (Fig 8A and 8A’) and *cxcl12b* (Fig 8B and 8B’) expression at 28 hpf, as shown by *in situ* hybridization. DEAB-treated embryos also display a strong increase in *meox1* expression at 28 hpf, when compared to controls (Fig 8C and 8C’). Combined, these data suggest that RA negatively regulates Cxcl12b-Cxcr4a pathway component gene expression at 28 hpf.

## Discussion

Previous research has shown that RA treatment of hematovascular precursors increases their ability to generate definitive hematopoietic precursors [16, 78], suggesting that RA signaling plays an instructive role in definitive hematopoiesis. This data is in line with previous analyses of RA function in mice, as *Aldh1a2*-mutants fail to correctly specify yolk sac hemogenic endothelial cells [14], and loss of *Aldh1a2* in VE-cadherin-positive endothelial cells is sufficient to abrogate HSC formation [16]. *Aldh1a2*-mutant mice die of severe vascular defects prior to HSC emergence [7], precluding global analyses of *Aldh1a2*-function in murine definitive hematopoiesis. We therefore used zebrafish as a model to study the role of RA signaling in definitive hematopoiesis.

Our study describes a novel role for RA signaling in definitive hematopoiesis. We propose that RA functions within the paraxial mesoderm or somites to regulate hematopoietic stem cell (HSC) formation. By impairing RA synthesis in the developing zebrafish embryo, we demonstrate that RA is required for proper HSC gene expression. In the absence of RA, embryos exhibit a severe reduction in HSC number and a corresponding failure to produce thymic lymphoid progenitors.

### RA regulates HSC formation independent of the Notch1-signaling pathway

Previous research in both mouse and zebrafish has established a model whereby Notch1-expressing cells within the dorsal aorta are instructed by adjacent cells to form HSCs [19, 20, 22–24]. *Notch1*-mutant embryonic stem cells fail to contribute to the wild type adult hematopoietic system in mouse chimeras [20], supporting this cell-autonomous role for Notch1 in definitive hematopoiesis. The yolk sac endothelial cells of *Aldh1a2*-mutant mice exhibit downregulated *Notch1* and Notch-target gene expression [59], implicating RA as a critical regulator of murine Notch1 signaling. Notch1 specifies HSCs [19, 20, 23, 24]. We, however, demonstrate that *notch1a/b* expression is unaffected by loss of RA in zebrafish. We further demonstrate that RA is required for HSC formation prior to the formation of dorsal aorta hemogenic endothelium. Our combined results therefore suggest that, unlike in mice, zebrafish RA does not regulate Notch1-signaling. We therefore propose a model whereby RA signaling acts outside of the pre-hemogenic endothelium, in a Notch1-independent fashion to regulate zebrafish HSC formation.

### RA may indirectly regulate Wnt16-Notch signaling

Recently, Clements et al., (2011) demonstrated a requirement for Wnt16 in zebrafish hematopoiesis. RA-deficient and *wnt16*-morphant embryos display common hematopoietic phenotypes; both demonstrate proper vascular gene expression and produce a functional dorsal aorta, but exhibit a severe reduction in HSC and common lymphoid progenitor gene expression [18]. These data suggest that both RA and Wnt16 are required for HSC formation. Our data, and previous results also suggest that both RA and Wnt16 function outside of dorsal aorta pre-hemogenic endothelium to regulate zebrafish HSC formation prior to 19 hpf [18]. Furthermore, both *aldh1a2* and *wnt16* are expressed in the paraxial mesoderm at this time [18]. In addition to hematopoietic defects, Wnt16-depleted embryos exhibit reduced somitic expression of the Notch ligands *dlc* and *dld* [18]. HSC gene expression is lost in *dlc*-mutants injected with *dld* morpholino, and *dlc*/*dld* overexpression rescues HSC gene expression in *wnt16*-morphants [18]. Notch3 is required by Wnt16-induced Dlc/Dld to regulate HSC formation [21]. We demonstrate that the expression of *notch3*, and its transcriptional target *her9* are not downregulated in RA-deficient embryos at 17 hpf. Our data therefore indicate that, despite their similar localization, and their common temporal requirement in definitive hematopoiesis, RA does not positively regulate *wnt16*, its downstream targets *dlc*, and *dld*, or *notch3*.

Dlc/Dld-mediated Notch signal transduction within pre-hematopoietic endothelial cells relies on the junctional adhesion molecules Jam1a/Jam2a [26]. We demonstrate that the anterior-most *jam1a*-expressing posterior lateral-plate mesoderm cells of RA-depleted embryos are improperly situated, and do not contact the somites. Furthermore, RA-depleted embryos exhibit upregulated, laterally expanded somitic *jam2a* expression. The expression domains of *jam1a/2a*, and the Notch transcriptional target *her9* do not significantly overlap at 17 hpf. Consequently, despite observing mildly upregulated *her9* expression in somitogenesis stage RA-depleted embryos, it remains possible that their *jam1a*-positive cell populations experience reduced Notch signaling. It is therefore currently unclear if the modifications to *jam1a*/*2a* expression that we observe in RA-depleted embryos are sufficient to disrupt Notch signaling within migrating pre-hematopoietic endothelial cells, or serve to reduce their definitive hematopoietic potential.

### RA differentially regulates the expression of early and late Cxcl12b signaling pathway components

Studies of Cxcl12b-signaling in zebrafish have revealed an essential role for this chemokine in definitive hematopoiesis. Targeted ablation of somitic *cxcl12b*-expressing endothelial cell precursors is sufficient to disrupt HSC formation in zebrafish, as is pharmacological or genetic inhibition of Cxcl12b signaling during somitogenesis stages [25]. We find that *cxcl12b* expression is subtly increased in the posterior somites of RA-depleted embryos, suggesting that RA may negatively regulate its expression.

*meox1*-null (*cho*) zebrafish mutants exhibit an increase in somitic *cxcl12b* expression, and a corresponding increase in HSC number [25]. Meox1-mediated chromatin immunoprecipitation of the zebrafish *cxcl12b* locus suggests that Meox1 is probably a direct inhibitor of *cxcl12b* [25]. We demonstrate that RA-depleted zebrafish embryos exhibit a subtle decrease in the posterior somitic expression of *meox1*. This decrease likely accounts for the increased *cxcl12b* expression that we observe in RA-depleted embryos. As these modifications to *meox1*/*cxcl12b* gene would be expected to generate increased HSC numbers [25], they do not explain the loss of HSCs that we observe in RA-depleted embryos.

Cxcl12b signaling occurs preferentially through the Cxcr4a receptor [79]. During zebrafish somitogenesis, *cxcr4a* is expressed within the anterior half of each somite (Fig 7A). We demonstrate that this expression is nearly abolished in RA-depleted embryos. Our data therefore implicates RA as a transcriptional regulator of *cxcr4a* within the somites. Given the requirement for Cxcl12b signaling in zebrafish definitive hematopoiesis, it is possible that the HSC gene expression defects that we observe in RA-depleted embryos may be partially attributable to reduced levels of *cxcr4a*. In support of this idea, we find that the RA-synthesis inhibitor DEAB and the Cxcr4-receptor antagonist AMD3100 act in concert to impair zebrafish embryonic HSC formation.

Lineage tracing experiments have shown that a proportion of *cxcl12b* and *cxcr4a*-expressing cells from the mediolateral portion of each somite colonize the dorsal aorta and dorsal-aorta supportive cells, but do not contribute to HSC populations directly [25]. This has led to the idea that Cxcl12b-signaling within the dorsal aorta may render endothelial cells competent to make HSCs [25]. Surprisingly, the early reduction of *cxcr4a* expression that we observe in RA-depleted embryos is not maintained; RA-depleted embryos exhibit strongly increased *cxcr4a*, *cxcl12b*, and *meox1* expression within the dorsal aorta and surrounding tissues at 28 hpf, just prior to HSC emergence (Fig 8A–8C’; S9 Table). Despite this increase, RA-depleted embryos do not produce HSCs. It is therefore possible that Cxcl12b/Cxcr4a signaling may regulate HSC formation earlier in development than previously thought (i.e. during somitogenesis). Alternatively, RA could act downstream of the Cxcl12b signaling pathway at 28 hpf, and *cxcl12b*/*cxcr4a* upregulation at this time may reflect the existence of some sort of negative-feedback loop. More stringent temporal analyses of Cxcl12b/Cxcr4a function in definitive hematopoiesis will be required to distinguish between these two possibilities.

Previous research has shown that *cxcr4a* expression is negatively regulated by hemodynamic force [65, 80], and that dorsal aorta *cxcr4a* expression is upregulated in embryos with reduced blood flow [80]. We observe mild circulatory and dorsal aorta morphology defects in a proportion of 2.5 μM DEAB-treated embryos at 28 hpf. It is therefore possible that the increased *cxcr4a* expression that we observe in these embryos results from decreased vascular perfusion. There are currently no published accounts linking hemodynamic force to changes in *cxcl12b* expression. It is not therefore clear if the upregulated *cxcl12b* expression that we observe in 28 hpf RA-depleted embryos might also be a consequence of impaired circulation.

The majority of *aldh1a2*-morphant and 2.5 μM DEAB-treated embryos exhibit normal posterior dorsal aorta and intersegmental vessel formation. Furthermore, although some *aldh1a2*-morphants exhibit delayed circulation, most possess visible circulating erythrocytes by 28 hpf. Our results further suggest that vascular and arterial gene expression is not altered in RA-depleted embryos. Nevertheless, RA-depleted embryos exhibit HSC gene expression defects. Our temporal analyses indicate that RA is required prior to the onset of dorsal aorta formation. Consequently, although previous studies have linked blood flow to HSC formation [52], our combined data suggest that the definitive hematopoietic phenotypes of RA-depleted embryos are not simply the consequence of reduced circulation or improper patterning of the dorsal aorta.

## Supporting Information

S1 Table Quantification of *cmyb* gene expression phenotypes in 32 hpf wild type (WT) controls and RA-deficient embryos. (DOCX)

S2 Table Quantification of thymic gene expression phenotypes in wild type (WT) and *aldh1a2*-morphant embryos. (DOCX)

S3 Table Quantification of circulatory phenotypes in wild type (WT) and *aldh1a2*-morphant embryos. (DOCX)

S4 Table Quantification of *Tg(kdrl*:*GFP)* dorsal aorta morphology phenotypes in 28 hpf wild type (WT) controls and RA-deficient embryos. (DOCX)

S5 Table Quantification of dorsal aorta gene expression phenotypes in wild type (WT) controls and RA-deficient embryos. (DOCX)

S6 Table Quantification of dorsal aorta *notch* gene expression phenotypes in 26 hpf wild type (WT) controls and RA-deficient embryos. (DOCX)

S7 Table Quantification of *cmyb* gene expression defects in 32 hpf wild type (WT) controls and *aldh1a2*-morphant embryos treated with 1 nM RA at indicated time points. (DOCX)

S8 Table Quantification of Wnt16-Notch3 pathway component gene expression phenotypes in 17 hpf wild type (WT) controls and RA-deficient embryos. (DOCX)

S9 Table Quantification of Chemokine pathway component gene expression phenotypes in 17 hpf wild type (WT) controls and RA-deficient embryos. (DOCX)
