# Correction: Somite-Derived Retinoic Acid Regulates Zebrafish Hematopoietic Stem Cell Formation

**DOI:** 10.1371/journal.pone.0171058

**Published:** 2017-01-25

**Authors:** Laura M. Pillay, Kacey J. Mackowetzky, Sonya A. Widen, Andrew Jan Waskiewicz

Figs 5 and 7 appear incorrectly in the published article. Please see the correct Figs 5 and 7 and their captions here.

**Fig 5:**
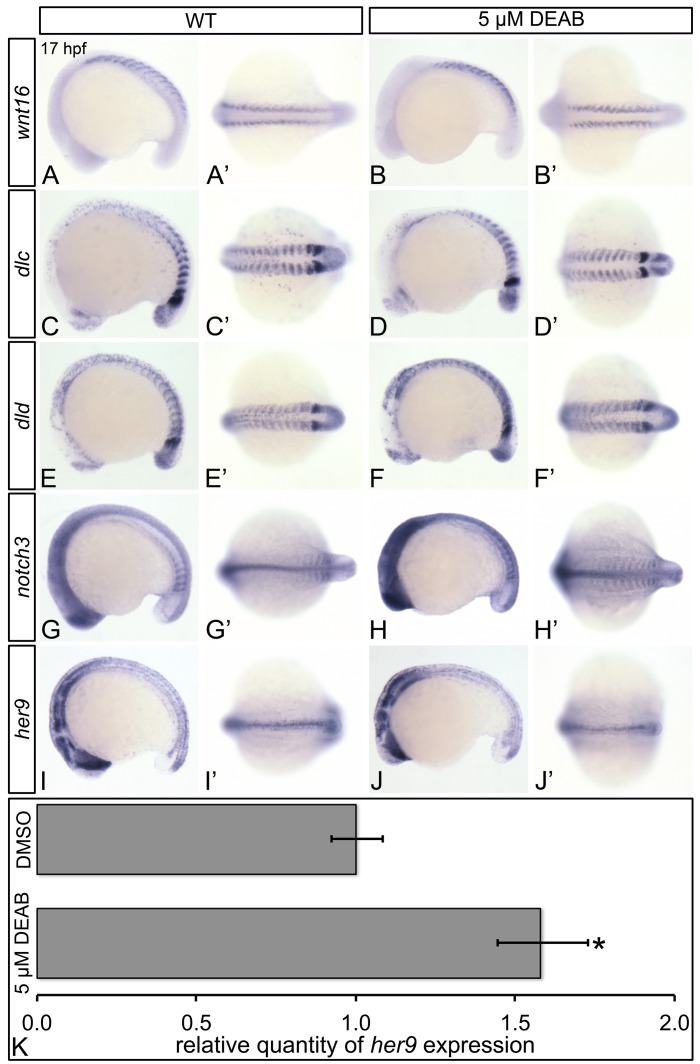
RA does not regulate the somitic expression of Wnt16-Notch3 signaling pathway components. Shown are representative 17 hpf embryos following *in situ* hybridization analyses (A-J’). Lateral view (A-J) or dorsal view (A’-J’) of gene expression is shown with anterior oriented to the left. A’-J’ represent different views of the embryos shown in A-J. Compared to DMSO-treated controls (A, A’, C, C’, E, E’, G, G’), DEAB-treated embryos exhibit normal somitic expression levels of *wnt16* (B, B’), and *dlc* (D, D’), mildly increased *dld* expression (F, F’), and increased *notch3* somitic gene expression (H, H’). DEAB-treated embryos also exhibit normal expression levels of the Notch3 signaling pathway transcriptional target *her9* (J, J’), when compared to DMSO-treated controls (I, I’). (K) Quantitative real-time PCR analysis of *her9* expression in 17 hpf DMSO-treated controls and embryos treated with 5 μM DEAB. Shown is the relative quantity of *her9* expression. Samples were normalized to *ef1a* and DMSO-treated was set to 1. Error bars indicate standard error of the mean. *Indicates the difference compared to control is significant by Student *t* test, *P* = 0.0198.

**Fig 7:**
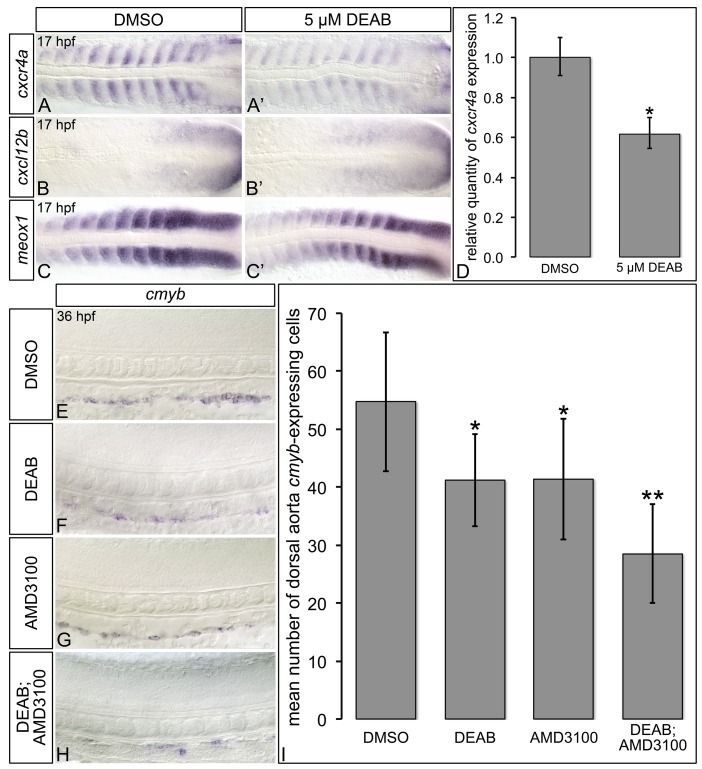
RA-deficient embryos exhibit altered Cxcl12b chemokine signaling pathway component gene expression. (A-C’) Representative flat-mount 17 hpf embryos following *in situ* hybridization analyses. Dorsal of gene expression is shown with anterior to the left. Compared to DMSO-treated controls (A), embryos treated with 5 μM DEAB (A’) exhibit strongly reduced somitic *cxcr4a* gene expression, and narrowing of the *cxcr4a* expression domain within each somite. Compared to DMSO-treated controls (B, C), embryos treated with 5 μM DEAB exhibit subtly increased levels of somitic *cxcl12b* expression (B’), and subtly decreased levels of somitic *meox1* expression (C’). (D) Quantitative real-time PCR analysis of *cxcr4a* expression in 17 hpf DMSO-treated controls and embryos treated with 5 μM DEAB. Shown is the relative quantity of *cxcr4a* expression. Samples were normalized to *ef1a* and DMSO-treated was set to 1. Error bars indicate standard error of the mean. *Indicates the difference compared to control is significant by Student *t* test, *P* < 0.0382. (E-H) Representative flat-mount 36 hpf embryos following *in situ* hybridization analyses of *cmyb* gene expression. Lateral view of gene expression in the dorsal aorta region of the trunk is shown with anterior to the left. Compared to DMSO-treated controls (E), embryos treated with 1 μM DEAB (F) or 10 μM AMD3100 (G) exhibit a small reduction *cmyb*-expressing cell numbers. Embryos treated with both 1 μM DEAB and 10 μM AMD310 (H) exhibit a more severe reduction in *cmyb*-expressing cell numbers. (I) Graph demonstrating the mean number of dorsal aorta *cmyb*-expressing cells in DMSO-treated controls, embryos treated with 1 μM DEAB, 10 μM AMD3100, or both 1 μM DEAB and 10 μM AMD310. Error bars represent standard error. *Indicates statistically significant difference compared to control (*P* ≤ 0.0144). **Indicates statistically significant difference compared to 1 μM DEAB, and 10 μM AMD3100 (*P* ≤ 0.0028). See text for statistical tests.
