# Supplementary material for: Somite-Derived Retinoic Acid Regulates Zebrafish Hematopoietic Stem Cell Formation
Source: PLoS One. 2016 Nov 18;11(11):e0166040. doi: 10.1371/journal.pone.0166040 (PMC5115706; doi:10.1371/journal.pone.0166040)
Supplement: Supplementary file 1 [file pone.0166040.s001.docx]

S1 Table. Quantification of *cmyb* gene expression phenotypes in 32 hpf wild type (WT) controls and RA-deficient embryos.

Treatment	Wild type	Reduced	Total	Two-tailed *P*-value
WT; DMSO	60	7	67	
aldh1a2 MO; DMSO	10	27	37	<0.0002*
5 µM DEAB	12	42	54	<0.0002*

*Indicates significant result compared to wild type (WT), by Fisher's Exact Test with Bonferroni correction on cumulative raw counts.
