# Supplementary material for: Somite-Derived Retinoic Acid Regulates Zebrafish Hematopoietic Stem Cell Formation
Source: PLoS One. 2016 Nov 18;11(11):e0166040. doi: 10.1371/journal.pone.0166040 (PMC5115706; doi:10.1371/journal.pone.0166040)
Supplement: Supplementary file 2 [file pone.0166040.s002.docx]

S2 Table. Quantification of thymic gene expression phenotypes in wild type (WT) and *aldh1a2*-morphant embryos.

Gene	Stage	Treatment	Wild type	Reduced	Total	Two-taile *P*-value
rag1	3 dpf	WT	65	7	72	
		aldh1a2 MO	14	31	45	<0.0001*
ikaros	3 dpf	WT	62	0	62	
		aldh1a2 MO	8	37	45	<0.0001*
foxn1	4 dpf	WT	45	2	47	
		aldh1a2 MO	43	3	46	0.6771

*Indicates significant result compared to wild type (WT), by Fisher's Exact Test on cumulativ raw counts.
