# Supplementary material for: Somite-Derived Retinoic Acid Regulates Zebrafish Hematopoietic Stem Cell Formation
Source: PLoS One. 2016 Nov 18;11(11):e0166040. doi: 10.1371/journal.pone.0166040 (PMC5115706; doi:10.1371/journal.pone.0166040)
Supplement: Supplementary file 3 [file pone.0166040.s003.docx]

S3 Table. Quantification of circulatory phenotypes in wild type (WT) and *aldh1a2*-morphant embryos.

Stage	Treatment	Circulation	No Circulation	Total	Mean % with Circulation	SEM (%)	Two-tailed *P*-value
28 hpf	WT	70	9	79	87.0	3.00	
	aldh1a2 MO	37	15	52	71.0	1.00	0.0196*
48 hpf	WT	74	1	75	97.0	3.00	
	aldh1a2 MO	48	2	50	96.5	3.50	0.5633

*Indicates significant result compared to wild type (WT), by Fisher's Exact Test on cumulative raw counts.
