# Supplementary material for: Somite-Derived Retinoic Acid Regulates Zebrafish Hematopoietic Stem Cell Formation
Source: PLoS One. 2016 Nov 18;11(11):e0166040. doi: 10.1371/journal.pone.0166040 (PMC5115706; doi:10.1371/journal.pone.0166040)
Supplement: Supplementary file 4 [file pone.0166040.s004.docx]

S4 Table. Quantification of *Tg(kdrl:GFP)* dorsal aorta morphology phenotypes in 28 hpf wild type (WT) controls and RA-deficient embryos.

Treatment	Mean % Wild type	SEM (%)	Mean % Thin	SEM (%)	Mean % Other	SEM (%)
WT; DMSO	100.0	0.0	0.0	0.0	0.0	0.0
2.5 µM DEAB	69.1	19.8	30.2	16.4	0.7	0.6
5 µM DEAB	19.1*	12.5	65.7*	13.7	15.2	1.2

*Indicates significant result ($P < 0.05$) compared to wild type (WT), by Bonferroni method post-test following two-way ANOVA. $F(4,18) = 11.18$, $P = 0.0001$.
