# Supplementary material for: Somite-Derived Retinoic Acid Regulates Zebrafish Hematopoietic Stem Cell Formation
Source: PLoS One. 2016 Nov 18;11(11):e0166040. doi: 10.1371/journal.pone.0166040 (PMC5115706; doi:10.1371/journal.pone.0166040)
Supplement: Supplementary file 5 [file pone.0166040.s005.docx]

S5 Table. Quantification of dorsal aorta gene expression phenotypes in wild type (WT) controls and RA-deficient embryos.

Gene	Stage	Treatment	Wild type	Reduced	Total	Two-taile *P*-value
kdrl	28 hpf	WT	16	2	18	
		aldh1a2 MO	14	3	17	0.6581
efnb2a	28 hpf	WT; DMSO	36	0	36	
		5 μM DEAB	37	1	38	1.000
