# Supplementary material for: Somite-Derived Retinoic Acid Regulates Zebrafish Hematopoietic Stem Cell Formation
Source: PLoS One. 2016 Nov 18;11(11):e0166040. doi: 10.1371/journal.pone.0166040 (PMC5115706; doi:10.1371/journal.pone.0166040)
Supplement: Supplementary file 6 [file pone.0166040.s006.docx]

S6 Table. Quantification of dorsal aorta *notch* gene expression phenotypes i
26 hpf wild type (WT) controls and RA-deficient embryos.

Gene	Treatment	Wild type	Abnormal	Total	Two-tail *P*-valu
notch1a	WT; DMSO	37	0	37	
	2.5 µM DEAB	49	0	49	1.000
notch1b	WT; DMSO	37	0	37	
	2.5 µM DEAB	58	1	59	1.000
notch3	WT; DMSO	78	2	80	
	2.5 µM DEAB	9	50	59	<0.0001
dlc	WT; DMSO	46	0	46	
	2.5 µM DEAB	56	0	56	1.000
her6	WT; DMSO	25	0	25	
	2.5 µM DEAB	24	0	24	1.000

*Indicates significant result compared to wild type (WT), by Fisher's Exact Test
cumulative raw counts.
