# Supplementary material for: Somite-Derived Retinoic Acid Regulates Zebrafish Hematopoietic Stem Cell Formation
Source: PLoS One. 2016 Nov 18;11(11):e0166040. doi: 10.1371/journal.pone.0166040 (PMC5115706; doi:10.1371/journal.pone.0166040)
Supplement: Supplementary file 7 [file pone.0166040.s007.docx]

[illegible] Table. Quantification of *cmyb* gene expression defects in [illegible] hpf wild type (WT) controls and *aldh1a2*-morphant embryos treated with 1 nM RA at indicated time points.

Treatment	Wild type	Reduced	Total	Two-tailed *P*-value
WT; DMSO	40	3	43	
WT; 1 nm RA at 4 hpf	35	2	37	1.000
aldh1a2 MO; DMSO	10	10	20	0.001*
aldh1a2 MO; 1 nM RA at 4 hpf	22	4	26	1.000
aldh1a2 MO; 1 nM RA at 19 hpf	7	23	30	<0.0005*
aldh1a2 MO; 1 nM RA at 24 hpf	6	19	25	<0.0005*

*Indicates significant result compared to wild type (WT), DMSO-treated controls by Fisher's Exact Test with Bonferroni correction on cumulative raw counts.
