# Supplementary material for: Somite-Derived Retinoic Acid Regulates Zebrafish Hematopoietic Stem Cell Formation
Source: PLoS One. 2016 Nov 18;11(11):e0166040. doi: 10.1371/journal.pone.0166040 (PMC5115706; doi:10.1371/journal.pone.0166040)
Supplement: Supplementary file 8 [file pone.0166040.s008.docx]

S8 Table. Quantification of Wnt16-Notch3 pathway component gene expression phenotypes in 17 hpf wild type (WT) controls and RA-deficient embryos.

Gene	Treatment	Wild type	Abnormal	Total	Two-tailed *P*-value
wnt16	WT; DMSO	43	0	43	
	5 µM DEAB	49	0	49	1.000
dlc	WT; DMSO	53	0	53	
	5 µM DEAB	55	0	55	1.000
dld	WT; DMSO	41	1	42	
	5 µM DEAB	9	25	34	<0.0001*
notch3	WT; DMSO	50	0	50	
	5 µM DEAB	4	36	40	<0.0001*
her9	WT; DMSO	40	0	40	
	5 µM DEAB	45	0	45	1.000
jam1a	WT; DMSO	65	0	65	
	5 µM DEAB	0	62	62	<0.0001*
jam2a	WT; DMSO	78	1	79	
	5 µM DEAB	4	65	69	<0.0001*

*Indicates significant result compared to wild type (WT), by Fisher's Exact Test on cumulative raw counts.
