# Supplementary material for: Somite-Derived Retinoic Acid Regulates Zebrafish Hematopoietic Stem Cell Formation
Source: PLoS One. 2016 Nov 18;11(11):e0166040. doi: 10.1371/journal.pone.0166040 (PMC5115706; doi:10.1371/journal.pone.0166040)
Supplement: Supplementary file 9 [file pone.0166040.s009.docx]

S9 Table. Quantification of Cxcl12b chemokine pathway component gene expression phenotypes in wild type (WT) controls and RA-deficient embryos.

Gene	Stage	Treatment	Wild type	Abnormal	Total	Two-tailed *P*-value
cxcr4a	17 hpf	WT; DMSO	48	5	53	
		5 μM DEAB	3	48	51	<0.0001*
cxcl12b	17 hpf	WT; DMSO	32	5	37	
		5 μM DEAB	19	24	43	<0.0001*
meox1	17 hpf	WT; DMSO	50	4	54	
		5 μM DEAB	12	40	52	<0.0001*
cxcr4a	28 hpf	WT; DMSO	47	0	47	
		2.5 μM DEAB	2	43	45	<0.0001*
cxcl12b	28 hpf	WT; DMSO	59	3	62	
		2.5 μM DEAB	49	42	91	<0.0001*
meox1	28 hpf	WT; DMSO	39	1	40	
		2.5 μM DEAB	6	35	41	<0.0001*

*Indicates significant result compared to wild type (WT), by Fisher's Exact Test on cumulative raw counts.
